# Children With Acute Lymphoblastic Leukemia in Romania: Results From a Decade-Long Single-Center Study

**DOI:** 10.7759/cureus.70166

**Published:** 2024-09-25

**Authors:** Alexandra Neaga, Laura Jimbu, Oana Mesaros, Cristina Blag, Mihnea Zdrenghea

**Affiliations:** 1 Department of Pediatric Oncology, Iuliu Hatieganu University of Medicine and Pharmacy, Cluj-Napoca, ROU; 2 Department of Hematology, Iuliu Hatieganu University of Medicine and Pharmacy, Cluj-Napoca, ROU; 3 Department of Hematology, Ion Chiricuta Oncology Institute, Cluj-Napoca, ROU; 4 Department of Mother and Child, Iuliu Hatieganu University of Medicine and Pharmacy, Cluj-Napoca, ROU; 5 Department of Pediatric Oncology and Hematology, Emergency Hospital for Children, Cluj-Napoca, ROU

**Keywords:** acute lymphoblastic leukemia, children, epidemiology, event-free survival, minimal residual disease, molecular biology, overall survival, risk group stratification

## Abstract

Background: Acute lymphoblastic leukemia (ALL) is the most prevalent cancer in children, with continuously improving survival rates. As few studies in Romania have analyzed ALL patients and disease characteristics or survival, we conducted a retrospective study on 158 patients diagnosed with ALL admitted to the Department of Pediatric Oncology and Hematology at the Emergency Hospital for Children, Cluj-Napoca, Romania, from January 2011 until April 2021. The most important objectives of the study are to establish full profiles of the patients and ALL, remission rates, relapses, and deaths, an epidemiology analysis to determine the incidence of ALL for comparison with the standard European population, and also to assess survival by the most important parameters, including minimal residual disease (MRD).

Methods: This was a retrospective study that focused on patients with newly diagnosed ALL from January 2011 to April 2021 in the Department of Pediatric Hematology and Oncology of the Emergency Hospital for Children, Cluj-Napoca, Romania. The inclusion criteria were: patients with de novo ALL, patients who were younger than 18 years of age, and patients with signed informed consents. The exclusion criteria included patients who were older than 18 years of age; patients with relapsed ALL; and patients who did not have a signed informed consent. A total of 158 patients were included in the study, aged between 0 and 17 years. The information about patient characteristics and all variables was taken from the patients' files, where informed consent is mandatorily stored. Descriptive statistics were used to evaluate variables such as age, gender, leukocyte number, immunophenotype, prednisone response, risk group assessment, cytogenetics, relapse, and MRD. Kaplan-Meier curves were used to assess survival, for which patients were divided into two subcohorts: a 2011-2015 subcohort and a 2016-2020 subcohort. The statistical analyses used Prism version 10.2.3 (GraphPad Software, La Jolla, CA). For the analysis, we used Kaplan-Meier curves, the log-rank (Mantel-Cox) test, the log-rank test for trends, the Gehan-Breslow-Wilcoxon test, survival proportions, the chi-square test, and correlations.

Results: A shift in risk groups was observed after the introduction of MRD testing in 2017, with more patients being stratified in the medium-risk group (MRG) and high-risk group (HRG). At the survival analyses between bone marrow (BM) aspiration and MRD on day 15, we discovered that patients with MRD>10% had much higher overall survival (OS) and event-free survival (EFS) compared to patients with >25% blasts in the BM; 24(11.4%) patients relapsed, of which, nine (3.8%) were very early relapses and 10 (4.1%) were late relapses. The five-year OS and EFS for patients with the T-cell immunophenotype of ALL and those with leukocyte counts >100,000/mm^3^ were identical to those at one year.

Conclusions: The OS of the 2016-2020 subcohort was higher than that of the 2011-2015 subcohort, and more patients were stratified into MRG and HRGs due to the implementation of MRD testing. Minimal residual disease testing helped to improve significantly the survival rates of patients with more than 10% residual disease. None of the patients with very early relapses entered complete remission (CR), but all late relapses achieved CR. All events experienced by patients with the T-cell immunophenotype, or leukocyte counts of >100,000/mm^3^ occurred in the first year after diagnosis.

## Introduction

Acute lymphoblastic leukemia (ALL) is the most common malignancy treated by pediatric oncologists, with approximately 80% of all pediatric cancers being diagnosed as ALL [1]. Despite the impressive results in achieving high complete remission (CR) rates in the last decades, with some current studies surpassing CR rates of 90%, nearly 20% of children relapse, and 15% surrender to the disease [2,3]. However, in low- and middle-income countries all over the world, survival rates vary between 50% and 75%, in part due to delays in diagnosis and treatment [4]. Usually, ALL is diagnosed in children between three and six years of age, with a higher frequency in males (male-to-female ratio = 1.4:1) [5]. Treatment is based on stratification of risk categories, which considers traditional risk factors according to international protocols, including age, white blood cell count (WBC), immunophenotype (B/T-cell lineage), cytogenetics, and response to therapy, and is best assessed by minimal residual disease (MRD). Minimal residual disease for tracing the leukemic clone is determined either with flow cytometry minimal residual disease (FCM-MRD) or with better sensitivity by polymerase chain reaction or next-generation sequencing and is a valuable tool for measuring response to therapy. Originally, MRD was rarely determined and only by international ALL study groups, but now most ALL protocols include MRD evaluation [5-7].

Acute lymphoblastic leukemia is a heterogeneous disease encompassing a constellation of dysregulated pathways that control cell proliferation, differentiation, and survival and play important roles in response to therapy [8]. Childhood ALL more frequently arises from B-cell rather than T-cell lineage. Acute lymphoblastic leukemia comprises cases correlated with hyperdiploidy, hypodiploidy, and recurrent chromosomal rearrangements leading to chimeric fusion genes. Fusion genes are highly related to therapy response, relapse, and prognosis [9]. Complex methods of detection determined that a pre-leukemic clone could lie quiescent for years and then become activated. Primary genetic defects are more consistent factors for prognosis compared to secondary genetic defects, which are usually copy number alterations (microdeletions and point mutations). t(12;21)/ETV6/RUNX1 is the most well-known biomarker in pediatric ALL and, together with hyperdiploidy, provides a good prognosis [5]. High-risk features of pediatric ALL include KMT2A (MLL) translocations, t(9;22)/BCR/ABL1, t(17;19)/TCF3-HLF, near haploidy and low haploidy, t(1;19)/TCF3-PBX1, intrachromosomal amplification of chromosome 21 (iAMP21), immunoglobulin heavy chains (IGH) translocations, Ikaros (IKZF1) gene deletions (a hallmark of pediatric high-risk ALL), and IKZF1plus (deletion without the ERG deletion but accompanied by other gene deletions such as CDKN2A/2B, PAX5, and PAR1) [5,10,11].

The aims of this study are to establish full profiles of patients diagnosed with ALL with the available data, response to treatment, moments of death, relapses, and achievement of secondary complete remission rates to characterize the cohort epidemiologically and the roles of MRD in risk group stratification and survival. We divided patients into two five-year subcohorts to assess survival by the most important variables, including MRD, to determine which factors influence survival.

## Materials and methods

Patients

This is a retrospective study that included patients with newly diagnosed ALL from January 2011 to April 2021 in the Department of Pediatric Hematology and Oncology of the Emergency Hospital for Children in Cluj-Napoca, Romania. The inclusion criteria were: patients with de novo ALL, patients who were younger than 18 years, and patients with signed informed consent. The exclusion criteria were patients who were older than 18 years; patients who relapsed ALL; and patients who did not have a signed informed consent. All legal guardians signed informed consent forms upon admission to our department, and the study was conducted in accordance with the Declaration of Helsinki. A total of 158 patients were included in the study, aged 0-17 years. The information about patient characteristics and all variables was taken from the patients' files, where the informed consent is mandatorily stored. Treatment was conducted according to the Acute Lymphoblastic Leukemia Intercontinental Berlin-Frankfurt-Munster Study Group 2002 (ALL IC-BFM 2002) protocol until May 2017 [12], when FCM-MRD was available; then treatment was administered according to the Acute Lymphoblastic Leukemia Intercontinental Berlin-Frankfurt-Munster Study Group 2009 (ALL IC-BFM 2009) [13].

Diagnosis

Peripheral blood (PB) samples, bone marrow (BM) aspirates, and lumbar punctures were performed at diagnosis and key moments during treatment. The diagnosis was based predominantly on cytological examination of the peripheral blood, morphological examination of the bone marrow smear (equal or more than 25% blasts), and immunophenotyping. Cerebrospinal fluid was analyzed for central nervous system (CNS) involvement. We determined the most frequent fusion genes in childhood ALL, but not all were available in our facility since 2011. Karyotyping was performed at diagnosis from BM aspirates.

Study variables

The study variables included gender, age at diagnosis, WBC, immunophenotype, risk group stratification, prednisone response on the eighth day of treatment, molecular biology, karyotype, BM morphology on days 15 and 33, and MRD analysis on days 15 and 33.

Risk group assessment was based on ALL IC-BFM 2002 from 2011 to 2017 and ALL IC-BFM 2009 from 2017 to 2021 and can be viewed in Tables 1-2 [12, 13]. 

**Table 1:** Risk group stratification according to the ALL IC-BFM 2002 protocol SRG: standard risk group; MRG: medium risk group; HRG: high-risk group; PB: peripheral blood; WBC: white blood cells; SR: standard risk; ALL IC-BFM 2002: Acute Lymphoblastic Leukemia Intercontinental Berlin-Frankfurt-Munster Study Group 2002 M1, M2, and M3 BM are defined as <5%, 5%-25%, and >25% blasts on the smear at the morphological examination, respectively Source: [12]

Criteria	SRG	MRG	HRG
PB on day 8	<1000 blasts/μL AND	<1000 blasts/μL AND	Equal or over 1000 blasts/μL
Age	Under or equal to 1 year and <6 years AND	<1 year and equal to or over 6 years AND/OR	All ages
Initial WBC count	<20000/μL AND	Equal or over 20000/μL AND	All WBC counts
BM morphology on day 15	M1or M2 marrow AND	M1 or M2 marrow AND	MRG and M3 marrow
BM morphology on day 33	M1 marrow	M1 marrow OR:	M2 OR M3 marrow
Translocations	-	-	t(9;22) BCR-ABL1 OR t(4;11) MLL-AF4
Observations	All conditions must be met	SR criteria, but M3 marrow on day 15 and M1 marrow on day 33	At least one condition must be met

**Table 2:** Risk group stratification according to ALL-IC BFM 2009 SRG: standard risk group; MRG: medium risk group; HRG: high-risk group; PB: peripheral blood; WBC: white blood cells; BM: bone marrow; FCM-MRD: flow-cytometry minimal residual disease; ALL IC-BFM 2009: Acute Lymphoblastic Leukemia Intercontinental Berlin-Frankfurt-Munster Study Group 2009 M1, M2, and M3 BM are defined as <5%, 5%-25%, and >25% blasts on the smear at the morphological examination, respectively Source: [13]

Criteria	SRG	MRG	HRG
PB on day 8	<1000 blasts/µL AND	<1000 blasts/µL​​​​​​​ AND	Equal to or over 1000 blasts/µL
Age	Under or equal to 1 year and <6 years AND	<1 year and equal to or over 6 years AND/OR	All ages
Initial WBC count	<20000/µL​​​​​​​ AND	Equal to or over 20000/mL AND	All WBC counts
BM morphology on day 15	M1or M2 marrow OR	M1or M2 marrow AND	MRG and M3 marrow OR
FCM-MRD on day 15	<0.1% (if available)	0.1%-10%	>10% (if available)
BM morphology on day 33	M1 marrow	M1 marrow	M2 OR M3 marrow
Translocations	-	-	t(9;22) BCR-ABL1 OR t(4;11) MLL-AF4
Karyotype			Hipodiploidy equal or under 44 chromosomes
Observations	All conditions must be met	Patients who do not have SRG or HRG criteria must be stratified in the MRG group	At least one condition must be met

M1, M2, and M3 BM are defined as <5%, 5%-25%, and >25% blasts on the smear at the morphological examination, respectively [13]. Prednisone good responders (PGR) on day eight were defined as <1000 blasts/mL in the PB, and prednisone poor responders (PPR) were defined as >1000 blasts/mL in the PB. All patients received seven days of prednisone treatment (60 mg/m2/day) prior to prednisone response evaluation and one administration of an intrathecal methotrexate dose (<1 year: 6 mg, one year: 8 mg, two years: 10 mg, equal or over three years: 12 mg), according to the protocol [13].

Complete remission before MRD availability was established with BM aspiration and was defined by M1 marrow on day 33 with no sign of extramedullary disease.

Minimal residual disease testing, which has been available in our facility since April 2017, was measured using a 10-color flow cytometer analyzer (Navios, Beckman Coulter, Brea, CA). Eight markers related to abnormal leukemia immunophenotypes were used, reaching sensitivity levels of 10-4. On day 15, MRD groups were defined as <0.1%, 0.1%-1%, 1%-10%, and >10%. On day 33, MRD groups were defined as <0.05% and >0.05% [13]. We started to determine MRD on day 33 in our department only since September 2019.

We recorded causes of death as "death in induction" and "death after induction", where the former describes death before day 33. We took into consideration toxic deaths due to infections and deaths due to progression or another comorbidity before day 33. Deaths after day 33 were due to treatment toxicity, very early, early relapse, late relapse, or due to another comorbidity. 

Key Concepts

As a key concept in this study, crude rate (incidence) defines the total number of certain health outcomes in a population without using specific aspects such as age, gender, or other variables referring to demography. Age-standardized rate (ASR) is used to compare the incidence of a disorder in different populations while considering differences in age distribution [14]. Event-free survival (EFS) is defined as the time from diagnosis to the last contact, which can indicate either CR or other events such as relapse, death, or secondary malignancy [15]. Overall survival (OS) is defined as the time from diagnosis to the last contact or death [15].

Data Analyses

Data analyses were performed using Prism version 10.2.3. (GraphPad Software, La Jolla, CA) and Microsoft Excel (Microsoft Corp., Redmond, WA). The epidemiologic study was achieved using crude rates and standardized rates. Crude rates were calculated with the formula number of cases/population*100,000, and ASRs were calculated using the standard European population from Eurostat (Appendix A) [16]. The formula used for ASR is the number of cases/standard population*100,000. The standard population was derived from the actual Romanian population divided by the standard European population weight (Table 1). The actual Romanian population by years and age groups was acquired from the Romanian National Institute of Statistics [16].

Descriptive statistics, tables, and charts were prepared using Microsoft Excel. Survival analysis and correlations between variables for the descriptive part of the study were done with Prism version 10.2.3. For the survival analysis, we used Kaplan-Meier curves, the log-rank (Mantel-Cox) test, the log-rank test for trends, the Gehan-Breslow-Wilcoxon test, the chi-square test, and survival proportions. A p-value of <0.05 was considered statistically significant.

## Results

Descriptive statistics

A total of 158 children with ALL were analyzed, and their characteristics are presented in Table 3. The cohort represents 15.3% of all the Romanian cases diagnosed with ALL during the described period (data extracted from the Romanian National Pediatric Onco-Hematology Registry [17]). Of the 158 patients, 78 (47.4%) were female and 86 (52.5%) were male, with a male-to-female ratio of 1.1/1. Eleven patients were lost from the study during diagnosis, therapy, or follow-up but were included in the study because we could find their survival status and date of death from the Directorate for the Registry of Persons and Database Management [18]. The median age at diagnosis was five years and six months (95% CI=4.97-6.26). with an age range of 0-17 years. Ninety-eight (62%) patients were between one and six years old, 55 (34.9%) were >6 years old, and only five (3.1%) were <1 year old.

**Table 3:** Characteristics of the study cohort (2011–2021)

Patient/disease characteristics	Variables	N %
Sex	Female	75 (47.4%)
	Male	83 (52.5%)
Age at diagnosis (years)	Median age	5.6 years
	<1	5 (3.1%)
	1–6	98 (62%)
	>6	55 (34.9%)
Leukocyte number /mL	<20000	95 (60.1%)
	20000–100000	44 (27.85)
	>100000	19 (12%)
Immunophenotype	B cells	134 (84.8%)
	T cells	24 (15.1%)
Karyotype	Normal	120 (75.9%)
	Hypodiploid	3 (1.9%)
	Hyperdiploid	8 (5%)
	Other anomalies	19 (12%)
Molecular biology	TEL-AML1/ETV6-RUNX1	45 (28.5%)
	E2A-PBX1/TCF3-PBX1	8 (5.1%)
	SIL-TAL1	10 (6.3%)
	BCR-ABL minor (p190)	7 (4.4%)
	MLL-AF4/KMT2A-AFF1	2 (1.3%)
	BCR-ABL major (p210)	1 (0.6%)
	No fusions	85 (53.8%)
Central nervous system-positive disease	>5 elements	9 (5.7%)
Prednisone response on day 8	Good response	127 (80.3%)
	Poor response	24 (15.2%)
Risk group	Standard risk group	36 (22.8%)
	Medium risk group	76 (48.1)
	High-risk group	38 (24%)
Bone marrow morphologic response on day 15	<5%	63 (40.9%)
	5-25%	31 (20.1%)
	³25%	21 (13.6%)
	Aplasia	36 (23.3%)
Bone marrow morphologic response on day 33	<5%	113 (78.5%)
	5-25%	8 (5.5%)
	³25%	1 (0.7%)
	Aplasia	22 (15.2%)
Flow-cytometry minimal residual disease on day 15 (from 2017)	<0.1%	20 (29.4%)
	0.1%–1%	17 (25%)
	1%–10%	17 (25%)
Flow-cytometry minimal residual disease on day 33 (from 2019)	<0.05%	14 (63.6%)
	>0.05%	8 (36.3%)

The median follow-up of the cohort was 1,319 days/44 months, with a range of 127 days/4.2 months to 3,730 days/124.3 months. Ninety-five (60.1%) patients had <20,000 leukocytes/µL at diagnosis, and 19 (12%) had >100,000 leukocytes/µL. The vast majority of children (134, 84.8%) had the B immunophenotype and only 24 (15.1%) had the T immunophenotype.

Regarding karyotype, 120 (75.9%) patients had normal karyotype, eight (5%) patients had hyperdiploidy, three (1.9%) patients had hypodiploid karyotype, and 19 (12%) had other anomalies, such as 47XX, +21, 45XX, +6, -2, 47XY, +8, 45XX, -9, inv9, del17, 45XY, -22, del8, del10, 45XX, -13, and -11. Forty-five (28.5%) patients had ETV6-RUNX1 translocation, and one patient (0.63%) had concomitant BCR-ABL1 major translocation. Eighty-five (53.8%) patients had no detectable fusion.

Only nine (5.7%) patients had CNS-positive disease. One hundred and twenty-seven (80.3%) patients were PGR on the eighth day, and 24 (15.2%) patients were PPR.

For BM morphologic response on day 15, 63 (40.9%) patients had M1 marrow, 31 (20.1%) had M2 marrow, and 21 (13.6%) had M3 marrow. As 36 (23.3%) patients had aplasia, their marrow response could not be evaluated at that time. For BM morphologic response on day 33, 113 (78.5%) patients had M1 marrow, eight (5.5%) had M2 marrow, and one (0.7%) had M3 marrow. As 22 (15.2%) patients had aplasia, their marrow response could not be evaluated at that time.

For those whose MRD was available on day 15 (54 patients, 34.1%), 20 (29.4%) achieved negative MRD (<0.1%), 17 (25%) had 0.1%-1% MRD, 17 (25%) had 1%-10% MRD, and 14 (20.6%) had >10% MRD. On day 33, FCM-MRD was negative for 14 (63.6%) patients and positive for eight (36.3%) patients.

**Figure 1:**
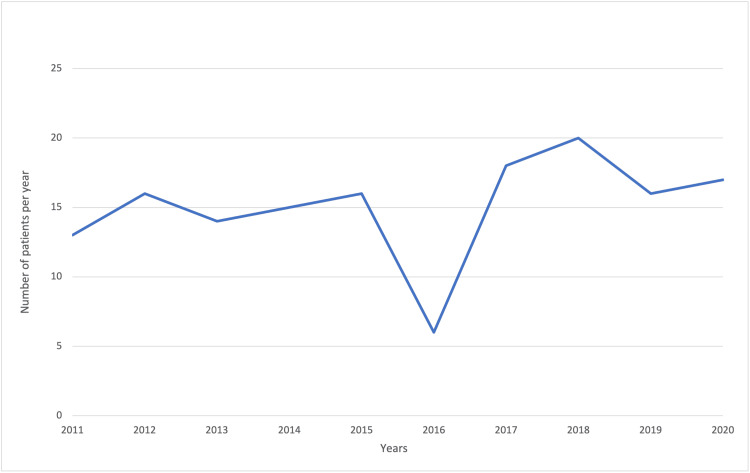
Number of cases of acute lymphoblastic leukemia per year for patients diagnosed between 2011 and 2021

The trend in the number of cases of ALL was approximately constant throughout the study period, with a drop in new cases in 2016 (Figure 1).

**Figure 2:**
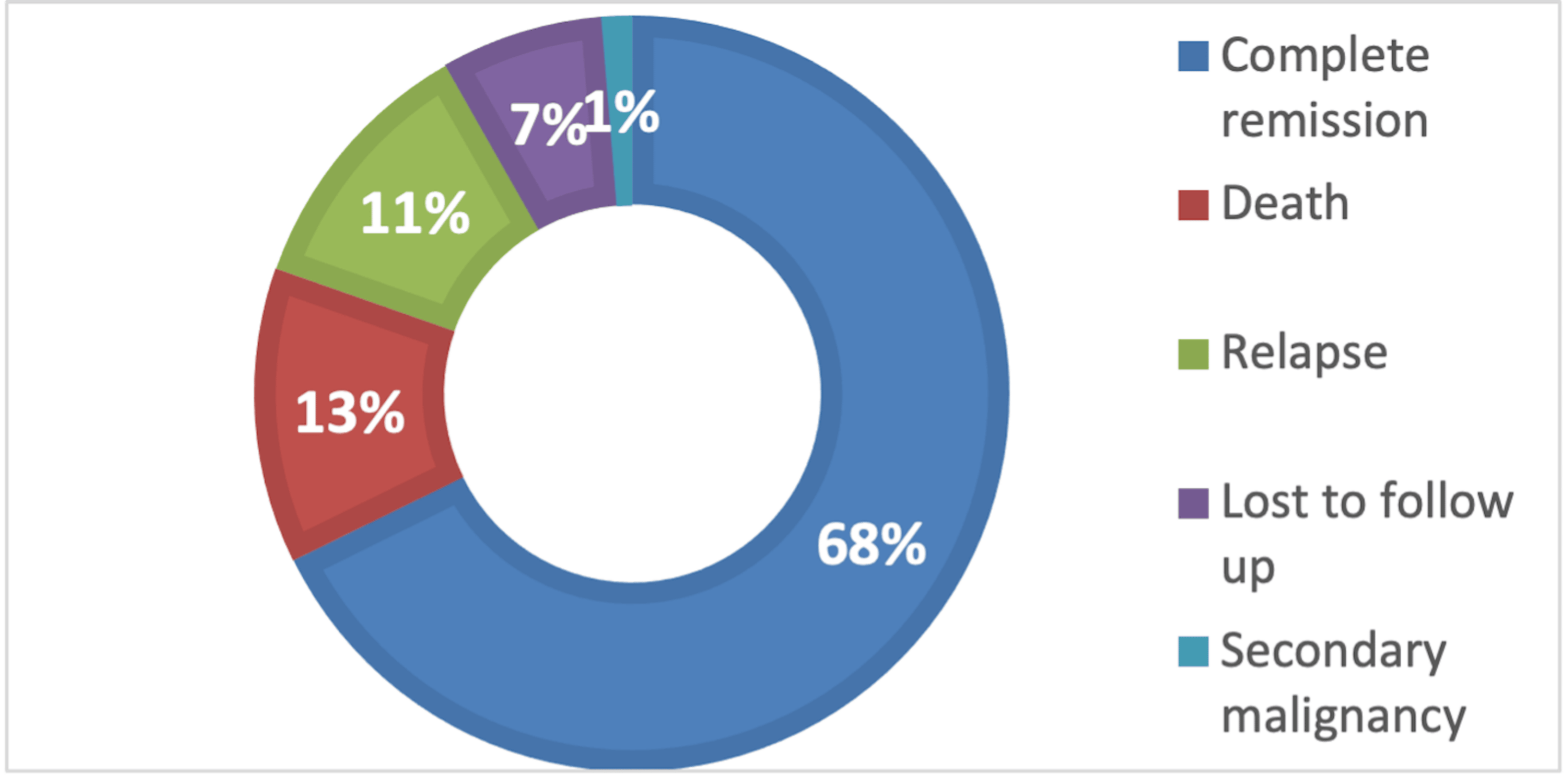
Outcomes of study cohort after the first line of chemotherapy

Within the study cohort, 107 (68%) patients obtained CR, 20 (13%) died, 18 (11%) relapsed, 11 (7%) were lost to follow-up, and two (1%) had a secondary malignancy (Figure 2).

**Figure 3:**
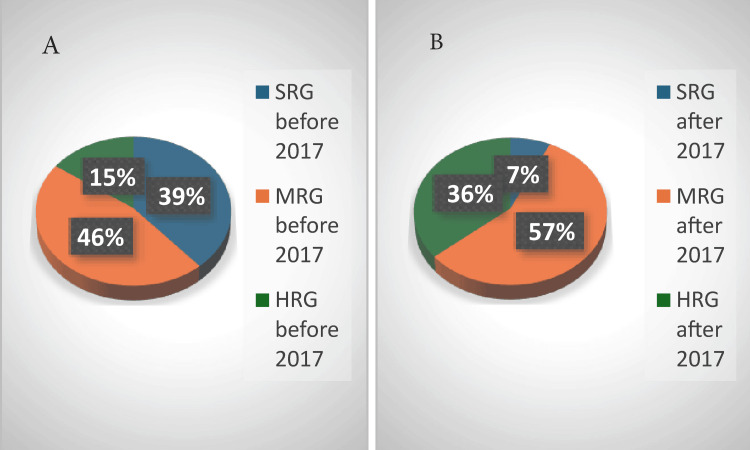
Risk groups (A) before and (B) after the introduction of minimal residual disease testing SRG: standard-risk group; MRG: medium-risk group; HRG: high-risk group

Before the introduction of MRD testing in 2017 (Figure 3), 61 (39%), 73 (46%), and 24 (15%) patients were stratified into the standard risk group (SRG), medium risk group (MRG), and high-risk group (HRG), respectively. After the introduction of MRD testing, only 11 (7%) patients could be stratified in the SRG, and the vast majority were stratified in the MRG and HRG at 90 (57%) and 57 (36%) patients, respectively. Referring to the time of treatment and the introduction of MRD testing, we observed that the same number of patients, 53 (33.7%), entered CR before and after 2017, but more patients relapsed (11, 7%) or died (17, 10.7%) before 2017 compared to after 2017, with seven (4.4%) relapses and six (5.4%) deaths. However, no statistically significant correlation was found (p = 0.29).

Epidemiology study

We analyzed the incidence rates of ALL per year for the entire cohort. To rule out confounding factors, we also calculated ASRs to account for uneven age distributions [19]. Over the entire study period, the crude rates and ASRs were almost identical for each year and close together between years. The lowest incidence was observed in 2016, and the highest incidence was observed in 2018 (Table 4).

**Table 4:** Incidence rates of ALL by year *Incidence reported for 100,000 population; **Median European Population (2016) [19] ASR: age-standardized rate; ALL: acute lymphoblastic leukemia

Year	Crude rate* (incidence)	ASR**
2011	0.31	0.31
2012	0.38	0.39
2013	0.34	0.35
2014	0.36	0.36
2015	0.40	0.40
2016	0.15	0.15
2017	0.44	0.44
2018	0.50	0.50
2019	0.39	0.40
2020	0.41	0.43

For epidemiological reasons, we stratified the patients into four groups according to age. The highest incidence rates were among children aged between 0 and four years, followed by those aged between five and nine years, and the lowest incidence rates were among those aged between 15 and 17 years (Table 5) [19].

**Table 5:** Incidence rates of ALL by age groups and year *Incidence reported for 100,000 population; **Median European Population (2016) [19] ASR: age-standardized rate; ALL: acute lymphoblastic leukemia

Age groups	0–4 years		5–9 years		10–14 years		15–17 years	
Year/Rates	Crude rate*	ASR**	Crude rate*	ASR**	Crude rate*	ASR**	Crude rate*	ASR**
2011	0.57	0.6	0.38	0.36	0.18	0.18	0.09	0.09
2012	0.97	1.0	0.38	0.36	0.18	0.18	0.00	0.00
2013	1.00	1.02	0.28	0.28	0.09	0.09	0.00	0.00
2014	0.52	0.51	0.75	0.75	0.19	0.19	0.00	0.00
2015	0.94	0.93	0.28	0.28	0.28	0.28	0.09	0.09
2016	0.32	0.31	0.28	0.28	0.00	0.00	0.00	0.00
2017	0.51	0.52	0.67	0.66	0.48	0.47	0.09	0.09
2018	1.10	1.15	0.6	0.57	0.19	0.19	0.09	0.09
2019	0.88	0.95	0.31	0.29	0.19	0.19	0.19	0.19
2020	1.06	1.16	0.21	0.19	0.19	0.19	0.19	0.19

Survival of the 2011-2015 subcohort

**Figure 4:**
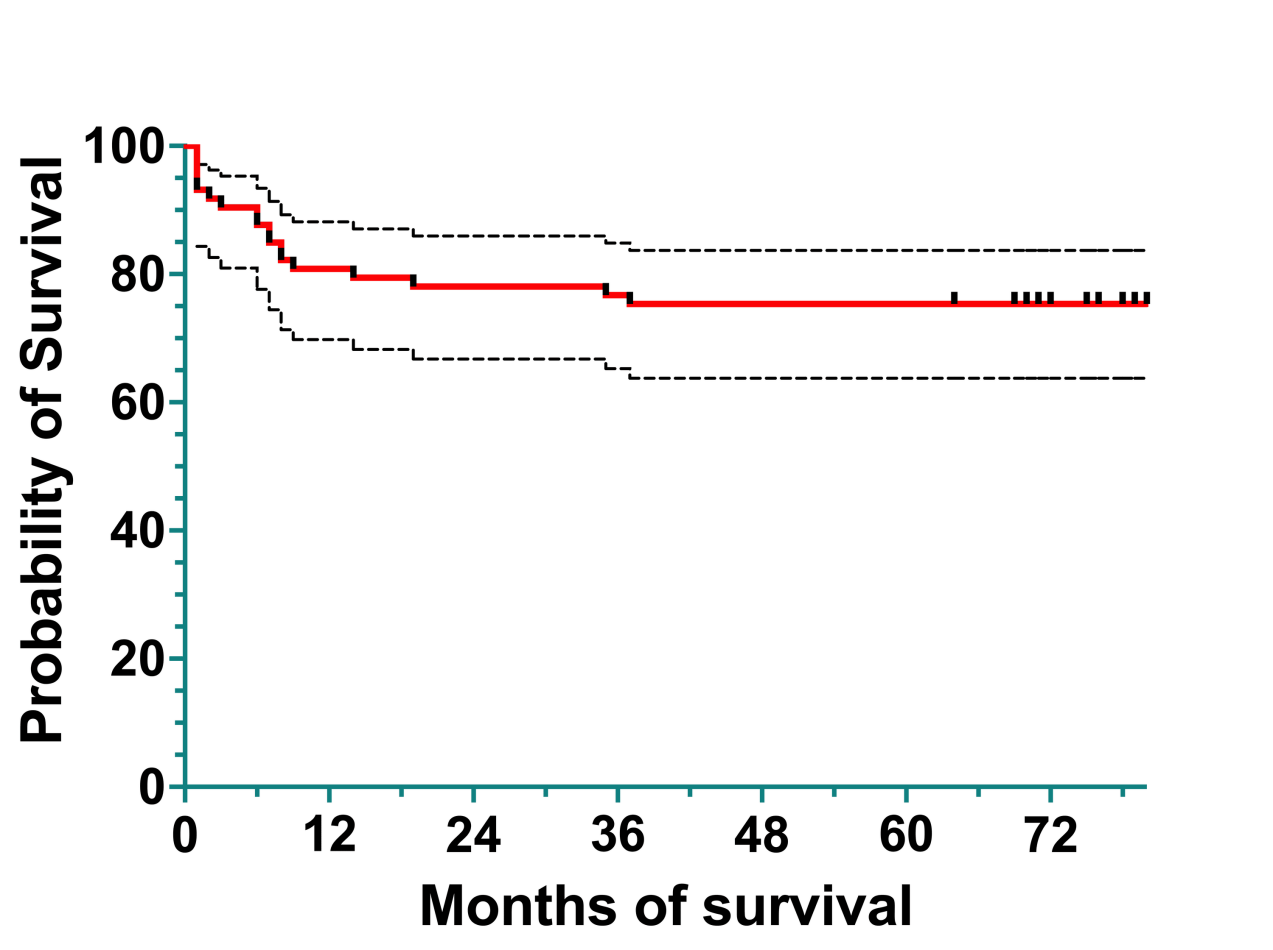
Overall survival at one, three, and five years

**Figure 5:**
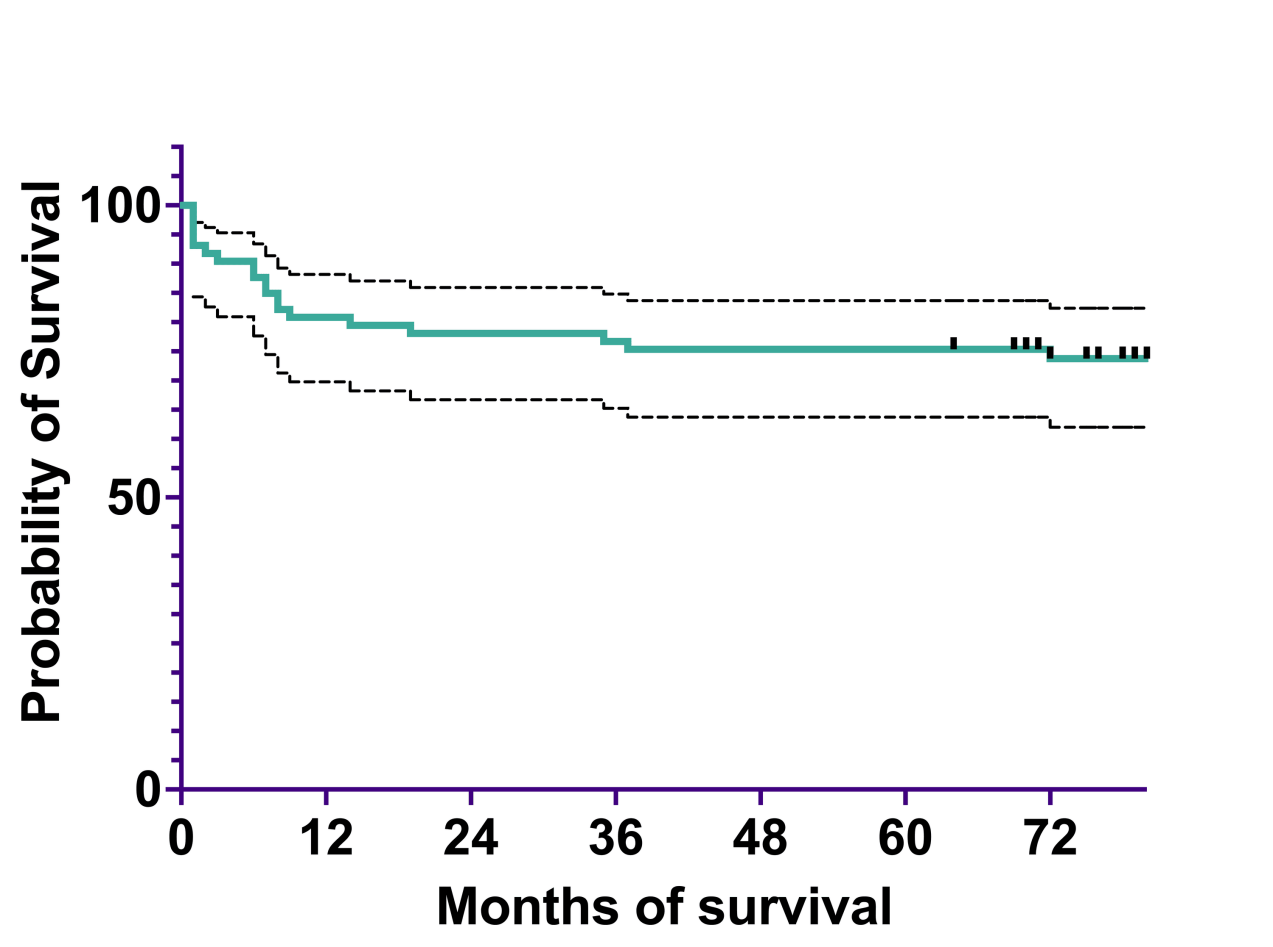
Event-free survival at one, three, and five years

To analyze survival, we divided the cohort into two subcohorts: a 2011-2015 subcohort and a 2016-2020 subcohort. The 2011-2015 subcohort included 74 patients diagnosed between January 1, 2011, and December 31, 2015. Overall survival was 60 (80.82%) patients at one year, 58 (78.08%) at three years, and 56 (75.34%) at five years. The EFS was 60 (80.82%) patients at one year, 57 (76.71%) at three years, and 56 (75.34%) at five years (Figures 4, 5).

The median survival was 79.5%, spanning 0 to 124 months (actual 95%CI=95.26), and the mean survival was 71.82% (95%CI=62.43-81.22).

The OS at one year was 50% (two) for infants <1 year of age, 85.71% (41) for children one to six years of age, and 77.77% (17) for children and adolescents >6 years of age (Table 6). The OS at three years was 50% (two) for infants <1 year of age, 80.95% (39) for children between one and six years of age, and 74.04% (16) for children and adolescents >6 years of age. At five years, OS remained 50% (two) for infants <1 year of age, 78.57% (39) for children between one and six years of age, and 74.07% (16) for children and adolescents >6 years of age.

**Table 6:** Survival of the 2011–2015 cohort across different variables The 2011-2015 subcohort had a follow-up period of five years, compared to the 2016-2020, which had a follow-up period of four years. For comparability, we added survival at four years in Table 6, and we observed that the differences in OS and EFS are minor. OR: odds ratio; OS: overall survival; EFS: event-free survival; 95%CI: 95% confidence interval; SRG: standard risk group; MRG: medium risk group; HRG: high risk group; PGR: prednisone good responders; PPR: prednisone poor responders

2011-2015 subcohort	OS at 1 year	95% CI	OS at 3 years	95%CI	OS at 4 years	95%CI	OS at 5 years	95%CI	p-value	EFS at 1 year	95% CI	EFS at 3 years	95%CI	EFS at 4 years	95%CI	EFS at 5 years	95%CI	p-value
	n%		n%		n%		n%			n%		n%		n%		n%		
	Survival of the 2011-2015 subcohort		80.82%		69,78-88.1		78.08%			66.74-85.95		75.34%		67-86.92		75.34%			63.76-83.68		80.82%	69.78-88.16	76.71%	69.78-88.16	75.34%	63.76-83.68	75.34%	63.76-83.68	
60		58	56	56		60	56	56	56																				
Survival by age groups									0.38									0.32
<1 year	50%	5.79-84.48	50%	5.79-84.48	50%	5.79-84.48	50%	5.79-84.48		60%	12.58-88.17	60%	12.58-88.17	60%	12.58-88.17	60%	12.58-88.17	
	2		2		2		2			2		2		2		2		
1-6 years	85.71%	70.94-93.31	80.95%	65.5-89.98	78.57%	68.92-94,27	78.57%	68.92-94.27		84.61%	68.96-92.77	79.31%	59.64-90.12	79.48%	69.8-95.82	76.93%	60.35-87.26	
	41		39		38		38			41		38		38		37		
>6 years	77.77%	50.78-89.33	74.04%	53.16-86.67	74.04%	53.16-86.67	74.07%	53.19-86.67		79.31%	59.64-90.12	75.86%	55.95-87.68	75.86%	55.95-87.68	75.86%	55.95-87.68	
	17		16		16		16			17		17		17		17		
Survival by immunophenotype									0.002									0.044
B cells	85.71%	74.35-92.29	80.95%	68.9-88.7	80.95%	68.9-88.7	79.36%	67.13-87.45		85.93%	74.72-91.41	81.25%	69.36-88.88	81.25%	69.36-88.88	79.68%	67.6-87.65	
	55		55		52		51			55		52		52		51		
T cells	44.44%	13.59-71.92	44.44%	13.59-71.92	44.44%	13.59-71.92	44.44%	13.59-71.92		44.4%	13.59-71.92	44.4%	13.59-71.92	44.4%	13.59-71.92	44.4%	13.59-71.92	
	4		4		4		4			4		4		4		4		
	Survival by risk groups																			0.0001									0.0001
SRG	96.42%	77.24-99.48	96.42%	77.24-99.48	96.42%	77.24-99.48	92.85%	74.35-98.15		96.49%	77.24-99.48	96.49%	77.24-99.48	96.49%	77.24-99.48	92.85%	74.35-98.15	
	27		27		27		26			27		27		27		26		
	MRG		87.09%		69.18-94.89		80.65%			61.92-90.79		80.64%		61.91-90.8		80.64%			61.91-90.8		87.09%	69.18-94.94	80.64%	61.91-90.97	80.64%	61.91-90.79	80.64%	61.91-90.79	
27		25	25	25		27	25	25	25																				
HRG	36.36%	11.18-62.67	27.27%	6.52-53.88	27.27%	6.52-53.88	27.27%	6.52-53.88		36.36%	11.18-62.67	27.27%	6.52-53.88	27.27%	6.52-53.88	27.27%	6.52-53.88	
	4		3		3		3			4		3		3		3		
Survival by gender									0.77									0.78
Females	80%	62.58-89.92	80%	62.58-89.92	77.14%	59.46-87.84	77.14%	59.46-87.84		80%	62.58-89.92	80%	62.58-89.92	77.14%	59.46-87.84	77.14	59.46-87.84	
	29		29		28		28			29		29		28		28		
Males	81.57%	65.2-90.75	73.68%	56.61-84.87	73.68%	56.61-84.87	73.68%	56.61-84.87		81.57%	65.2-90.75	73.68%	56.61-84.87	73.68%	56.61-84.87	73.68%	56.61-84.87	
	31		28		28		28			31		28		28		28		
Survival by prednisone response									0.02									0.06
PGR	85.24%	73.56-92.02	81.96%	69.81-89.57	80.32%	67.96-88.30	80.32%	67.96-88.30		85.24%	73.56-92.02	81.96%	69.81-89.57	80.32%	67.96-88.30	80.32%	67.96-88.30	
	52		50		49		49			52		50		49		49		
PPR	58.33%	27.02-80.09	50%	20.85-73.60	50%	20.85-73.60	50%	20.85-73.60		58.33%	27.02-80.09	58.33%	27.02-80.09	58.33%	27.02-80.09	58.33%	27.02-80.09	
	6		5		5		5			6		6		6		6		
Survival by leukocyte count									0.008									0.003
L<20,000/µL	86.95%	73.25-93.91	86.95%	73.25-93.91	84.78%	70.71-92.43	84.78%	70.71-92.43		88.63%	74.83-95.09	88.63%	74.83-95.09	86.36 %	72.14-93.62	86.36 %	72.14-93.62	
	40		40		39		39			41		41		40		40		
L 20,000/µL -100,000/µL	82.35%	54.72-93.93	64.70%	37.71-82.33	64.70%	37.71-82.33	64.70%	37.71-82.33		83.33%	56.77-94.29	66.66%	40.34-83.42	66.66%	40.34-83.42	66.66%	40.34-83.42	
	14		11		11		11			14		11		11		11		
L>100,000/µL	50%	18.37-75.31	50%	18.37-75.31	50%	18.37-75.31	50%	18.37-75.31		45.45%	16.66-70.68	45.45%	16.66-70.68	45.45%	16.66-70.68	45.45%	16.66-70.68	
	5		5		5		5			5		5		5		5		

The EFS at one year for infants <1 year of age was 60% (two), 84.61% (41) for children between one and six years of age, and 79.31% (17) for children and adolescents >6 years of age. At three years, EFS was the same: 60% (two) for infants <1 year of age, 79.48% (38) for children between one and six years of age, and 75.86% (17) for children and adolescents >6 years of age. At five years, EFS was the same as at three years, 60% (two), for infants <1 year of age, 76.93% (37) for children between one and six years of age, and the same as at three years, 75.86% (17), for children and adolescents >6 years of age.

At one year, OS by immunophenotype was 85.71% (55) for the B immunophenotype and 44.44% (four) for the T immunophenotype (Table 6). At three years, OS was 80.95% (52) for the B immunophenotype, the same as that at one year, 44.44% (four) for the T immunophenotype. At five years, OS was 79.36% (51) for the B immunophenotype and the same as that at one and three years, 44.44% (four) for the T immunophenotype. The OS survival curves for the two immunophenotypes had statistically significant differences (p = 0.002).

At one year, EFS was 85.93% (55) for the B immunophenotype and 44.4% (4) for the T immunophenotype. At three years, EFS was 81.25% (52) for the B immunophenotype and the same as that at one year, 44.4% (four), for the T phenotype. At five years, EFS was 79.68% (51) for the B immunophenotype, and the same as that at one and three years, 44.44% (four) for the T immunophenotype. This difference in EFS between groups was determined to be statistically significant (p = 0.044).

Considering the risk group stratification, the differences in OS and EFS between groups were determined to be statistically significant (p = 0.002 and p = 0.001, respectively). At one year, OS was 96.42% (27) for the SRG, 87.09% (27) for the MRG, and 36.36% (four) for the HRG (Table 6). At three years, OS was the same as that at one year: 96.42% (27), 80.65% (25) for the SRG, and 27.27% (three) for the HRG. At five years, OS was 92.85% (26) for the SRG, 80.64% (25) for the MRG, and the same as that at three years, 27.27% (three) for the HRG.

At one year, EFS was 96.49% (27) for the SRG, 87.09% (27) for the MRG, and 36.36% (4) for the HRG. At three years, EFS was the same as that at one year: 96.49% (27), for the SGR, 80.64% (25) for the MRG, and 27.27% (three) for the HRG patients. At five years, EFS was 92.85% (26) for the SRG, 80.64% (25) for the MRG, and the same as that at three years, 27.27% (three) for the HRG.

Referring to the gender of the patients, at one year, OS was 80% (29) for females and 81.57% (31) for males (Table 6). At three years, OS was the same as that at one year: 80% (29), for females, and 73.68% (28) for males. At five years, OS was 77.14% (28) for females and 73.68% (28) for males.

At one year, EFS was 80% (29) for females and 81.57% (31) for males. At three years, EFS was the same as that at one year: 80% (29), for females, and 73.68% (28) for males. At five years, EFS was 77.14% (28) for females and the same as that at three years, 73.68% (28), for males.

Looking at prednisone response, we found that at one year, OS was 85.24% (52) for the PGR group and 58.33% (six) for the PPR group (Table 6). At three years, OS was 81.96% (50) for the PGR group and 50% (5) for the PPR group. At five years, OS was 80.32% (49) for the PGR group and the same as that at three years, 50% (six), for the PPR group. This difference in OS between groups was determined to be statistically significant (p = 0.02).

At one year, EFS was 85.24% (52) for the PGR group and 58.33% (six) for the PPR group, with the latter remaining unchanged at three and five years. At three years, EFS was 81.96% (50) for the PGR group. At five years, EFS was 80.32% (49) for the PGR group.

Considering the number of leukocytes at diagnosis, we found that at one year, OS was 86.95% (40) for those with leukocyte counts of <20,000/µL, 82.35% (14) for those with leukocyte counts of 20,000-100,000/µL, and 50% (five) for those with leukocyte counts >100,000/µL (Table 6). At three years, OS was the same as that at one year, 86.95% (40), for the <20,000/µL group, and 64.70% (11) for the 20,000-100,000/µL group, with the latter remaining the same at five years; the >100,000/µL group had the same OS as that at one year and five years. At five years, OS was 84.78% (39) for the <20000/µL group.

At one year and three years, EFS was 88.63% (41) for the <20,000/µL group. In the first year, EFS was 83.33% (14) for the 20,000-100,000/µL group and 45.45% (five) for the >100,000/µL group. At three and five years, EFS was 66.66% (11) for the 20,000-100,000/µL group and 45.45% (five) for the >100,000/µL group. At five years, EFS was 86.36% (40) for the <20,000/µL group. These differences in OS and EFS between groups were determined to be statistically significant (p = 0.008 and p = 0.003, respectively).

Survival of the 2016-2021 subcohort

The 2016-2020 cohort included 77 patients diagnosed with ALL between January 1, 2016, and December 31, 2020, with a follow-up period of four years. Survival at five years will be available for verification in the year 2025. For comparability, we added survival at four years in Table 7, and we observed that the differences in OS and EFS are minor.

**Table 7:** Survival of 2016–2020 cohort across different variables OS: overall survival; EFS: event-free survival; 95%CI: 95% confidence interval; SRG: standard risk group; MRG: medium risk group; HRG: high risk group; PGR: prednisone good responders; PPR: prednisone poor responders

Cohort 2016–2020	OS at one year n %	95% CI	OS at three years n %	98% CI	OS at four years n %	95% CI	p-value	EFS at one year n %	95% CI	EFS at three years n %	95% CI	EFS at four years n %	95% CI	p-value
Survival of the entire 2016– 2020 cohort	86.33% 66	76.02– 92.42	80.56% 62	68.56– 88.35	76.73% 59	62.29– 86.22		86.14% 66	75.71– 92.31	75.44% 58	61.65– 84.85	64.66 % 50	44.17– 79.24	
Survival by age groups							0.01							0.008
<1 year	0		0		0			0		0		0		
1–6 years	85.17% 37	69.74– 93.09	85.17% 37	69.74– 93.09	85.17% 37	69.74– 93.09		85.17% 37	69.74– 93.09	85.17% 37	69.74– 93.09	70.97% 31	35.32– 89.31	
>6 years	87.13% 28	69.20– 94.97	87.13% 28	69.20– 94.97	75.41% 22	54.47– 87.55		83.91% 27	65.53– 92.97	64.95% 21	44.17– 79.61	42.63% 14	18.28– 65.20	
Survival by immunophenotype							0.19							0.29
B cells	85.57% 55	74.06– 92.23	79.25% 51	66.10– 87.75	75.29% 48	59.91– 85.45		83.93% 54	72.15– 91.02	75.96% 49 s	62.59– 85.10	52.96% 34	32.22– 69.72	
T cell	62.30% 8	27.35– 84.13	62.30% 8	27.35– 84.13	62.30% 8	27.35– 84.13		62.30% 8	27.35– 84.13	62.30% 8	27.35– 84.13	62.30% 8	27.35– 84.13	
Survival by risk groups							0.32							0.1
SRG	77.77% 6	36.47– 93.92	77.77% 6	36.47– 93.92	77.77% 6	36.47– 93.92		77.77% 6	36.47– 93.92	77.77% 6	36.47– 93.92	77.77% 6	36.47– 93.92	
MRG	89.57% 36	74.42– 95.95	86.37% 35	70.14– 94.12	79.72% 32	57.33– 91.23		89.57% 36	74.52– 95.95	80.20% 32	58.68– 91.27	51.82% 21	20.99– 75.81	
HRG	77.94% 20	54.3– 90.32	65.95% 17	40.58– 82.52	65.95% 17	40.58– 82.52		73.07% 19	49.13– 87.06	57.41% 15	33.59– 74.40	45.93% 12	19.53– 69.02	
Survival by gender							0.37							0.15
Females	82.35% 30	64.75– 91.68	70.17% 25	49.82– 83.51	70.17% 25	49.82– 83.51		82.35% 30	64.75– 91.68	67.12% 24	46.97– 81.03	40.27% 14	15.72– 63.94	
Males	84.47% 35	68.56– 92.73	84.47% 35	100– 91.69	77.97% 32	56.38– 89.75		81.91% 35	65.69– 90.95	76.45% 31	56.69– 88.06	70.57% 29	48.47– 84.55	
Survival by prednisone response							0.03^*^							0.06
PGR	86.54% 53	74.84– 93.04	82.20% 50	69.24– 90.07	78.29% 48	62.95– 87.86		84.87% 52	72.91– 91.83	75.60% 46	61.07– 85.33	56.41% 34	35.60– 72.80	
PPR	66.12% 9	31.56– 86.20	52.89% 7	19.02– 78.31	52.89% 7	19.02– 78.31		66.12% 9	31.56– 86.20	52.89% 7	19.02– 78.31	52.89% 7	19.02– 78.31	
Survival by leukocyte count							0.3							0.31
<20,000/µL	86.05% 39	71.48– 93.49	79.89% 36	63.41– 89.5	72.67% 33	50.27– 86.18		86.05% 39	71.48– 93.49	79.89% 36	63.41– 89.52	51.87% 23	23.01– 74.58	
20,000–100,000/ µL	86.66% 21	63.99– 95.51	80.47% 19	55.26– 92.34	80.47% 19	55.26– 92.34		82.33% 20	59.44– 92.99	64.50% 15	38.49– 81.75	53.75% 13	25.24– 75.58	
>100,000/µL	58.33% 5	18.02– 84.40	58.33% 5	18.02– 84.40	58.33% 5	18.02– 84.40		58.33% 5	18.02– 84.4	58.33% 5	18.02– 84.4	58.33% 5	18.02– 84.4	

**Figure 6:**
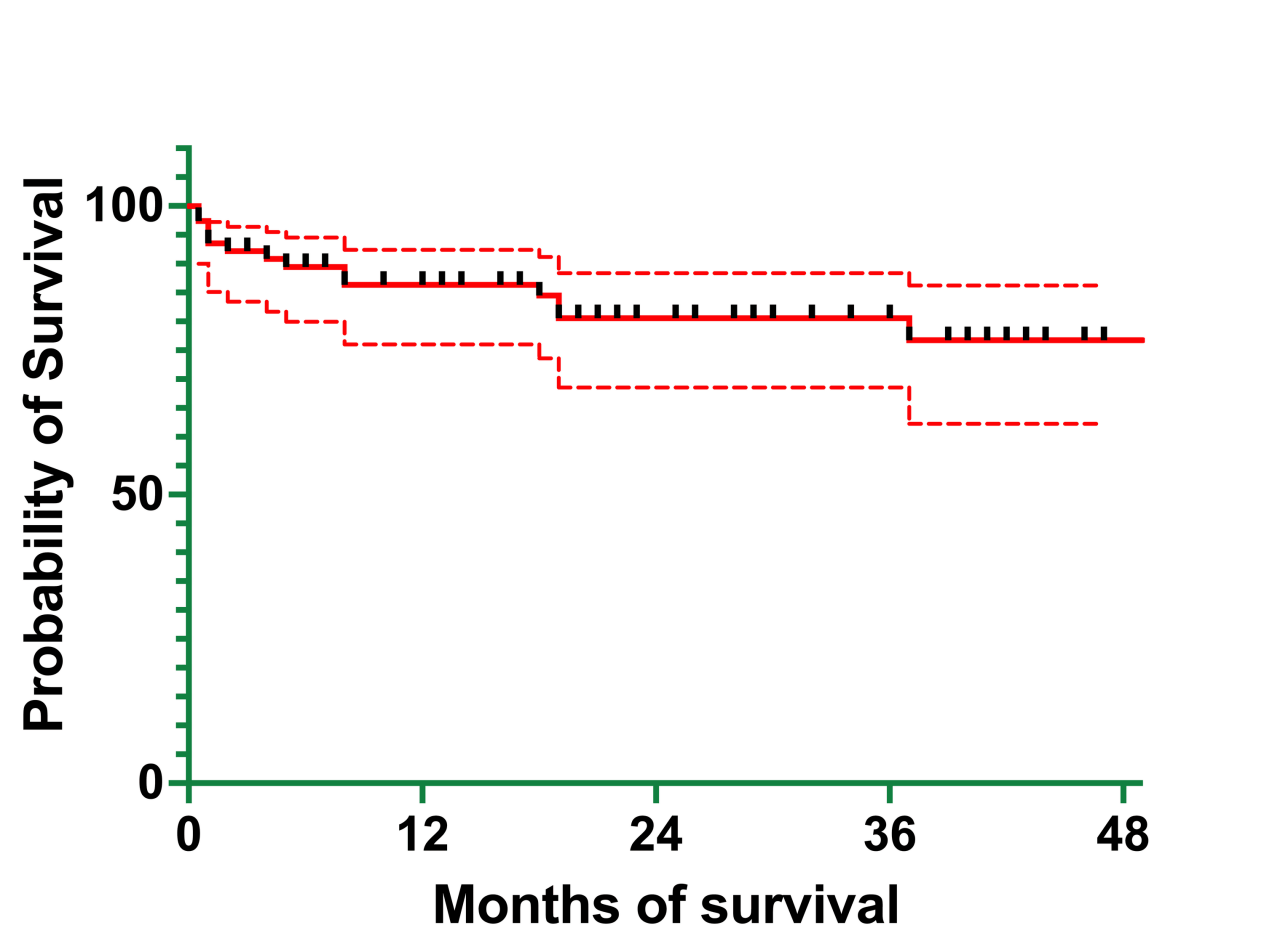
Overall survival of the 2016–2020 cohort

**Figure 7:**
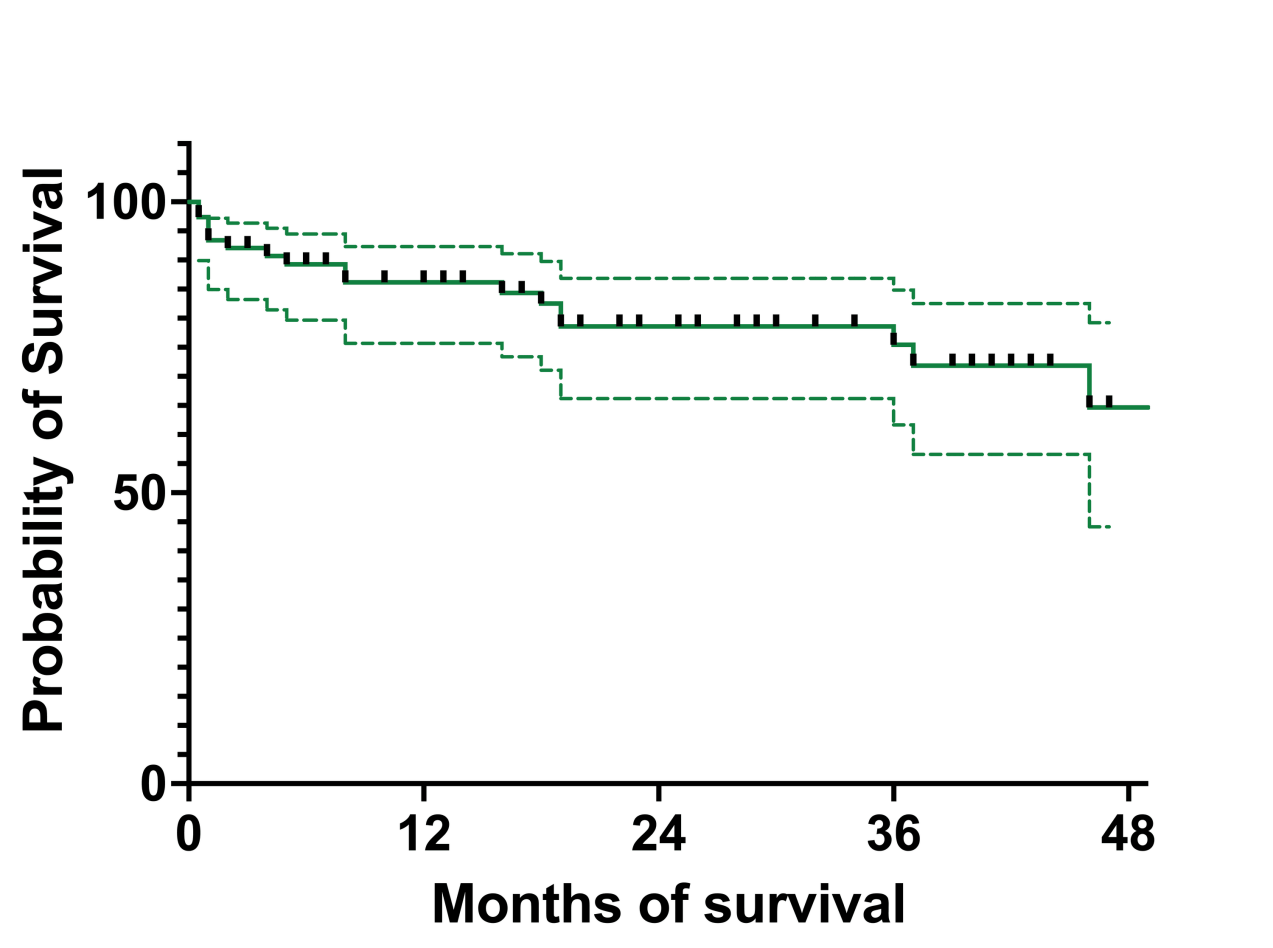
Event-free survival of the 2016–2020 cohort

The OS of the cohort was 86.33% (66) at one year, 80.56% (62) at three years, and 76.73% (59) at four years (Figure 6). The EFS of the cohort was 86.14% (66) at one year, 75.44% (58) at three years, and 64.66% (50) at four years (Figure 7). The median survival was 21 months, with ranges between 0.5 and 62 months (actual 95% CI = 96.05). The mean survival was 23.97 months (95% CI=20.05-27.90).

At one year, OS was 0% (one patient who died at five months of age) for infants <1 year of age, 85.17% (37) for children aged between one and six years, and 87.13% (28) for children and adolescents >6 years of age, the same percentage recorded at three years (Table 7). At three years, OS was the same as it was at one year and four years for the children in the one-to-six-year age group and 75.41% (24) for the >6-years group. At four years, OS was 69.61% (22) for the >6-years group.

At one year, EFS was 0% (one patient) for the <1-year group, 85.17% (37) for the one- to six-year group, the same as the one at three years, and 83.91% (27) for the >6-years group. At three years, EFS was 64.95% (21) for the >6-years group. At four years, EFS was 70.97% (31) for the children in the one- to six-year age group and 42.63% (14) for the >6-year age group. These differences in OS and EFS between groups were determined to be statistically significant (p = 0.01 and p = 0.008, respectively).

At one year, OS was 85.57% (55) for patients with the B immunophenotype of ALL and 62.30% (8) for those with the T immunophenotype of ALL, the same as that at three and four years (Table 7). At three years, OS was 79.25% (51) for patients with the B immunophenotype, and at four years it was 75.29% (48).

At one year, EFS was 83.93% (54) for those with the B immunophenotype and 62.30 (eight) for those with the T immunophenotype, the same as at three and four years. At three years, EFS was 75.96% (49) for those with the B immunophenotype, and at four years it was 52.96% (34).

At one year, OS was 77.77% (six) for the SRG, which was the same at three years and four years, 89.57% (36) for the MRG group, and 77.94% (20) for the HRG (Table 7). At three years, OS was 86.37% (35) for the MRG and 65.95% (17) for the HRG. At four years, OS was the same as at three years for the HRG and 79.72% (32) for the MRG.

At one year, EFS was 77.77% (six) for the SRG, the same as OS at one year, which remained unchanged at three and four years: 89.57% (36) for the MRG, and 73.07% (19) for the HRG. At three years, EFS was 80.20% (32) for the MRG and 57.41% (15) for the HRG. At four years, EFS was 51.82% (21) for the MRG and 45.93% (12) for the HRG.

At one year, OS was 84.47% (35) for males and 82.35% (30) for females (Table 7). At three years, OS was 84.47% (35) for males and 70.17% (25) for females, which remained unchanged at four years. At four years, OS was 77.97% (32) for males.

At one year, EFS was 81.91% (34) for males and 82.35% (30) for females. At three years, EFS was 76.45% (31) for males and 67.12% (24) for females. At four years, EFS was 70.57% (29) for males and 40.27% (14) for females.

At one year, OS was 86.54% (53) for the PGR group and 66.12% (nine) for the PPR group (Table 7). At three years, OS was 82.20% (50) for the PGR group and 52.89% (seven) for the PPR group, the same as at four years. At four years, OS was 78.29% (48) for the PGR group.

At one year, EFS was 84.87% (52) for the PGR group and 66.12% (nine) for the PPR group. At three years, EFS was 75.60% (46) for the PGR group and 52.89% (seven) for the PPR group, the same as at four years. At four years, EFS was 56.41% (34) for the PGR group. This difference in OS between groups was determined to be statistically significant (p = 0.03).

At one year, OS was 86.05% (39) for patients who had leukocyte counts of <20,000/µL, 86.66% (21) for those with leukocyte counts of 20,000-100,000/µL, and 58.33% (five) for those with leukocyte counts of >100,000/µL, the same as that at three and four years (Table 7). At three years, OS was 79.89% (36) for the <20,000/µL group and 80.47% (19) for the 20,000-100,000/µL group, the same as that at four years. At four years, OS was 72.67% (33) for the <20,000/µL group.

At one year, EFS was 86.05% (39) for the <20,000/µL group, 82.33% (20) for the 20,000-100,000/µL group, and 58.33% (5) for the >100,000/µL group, the same as that at three and four years. At three years, EFS was 79.89% (36) for the <20,000/µL group and 64.50% (15) for the 20,000-100,000/µL group. At four years, EFS was 51.87% (23) for the <20000/µL group and 53.75% (13) for the 20,000-100,000/µL group.

**Figure 8:**
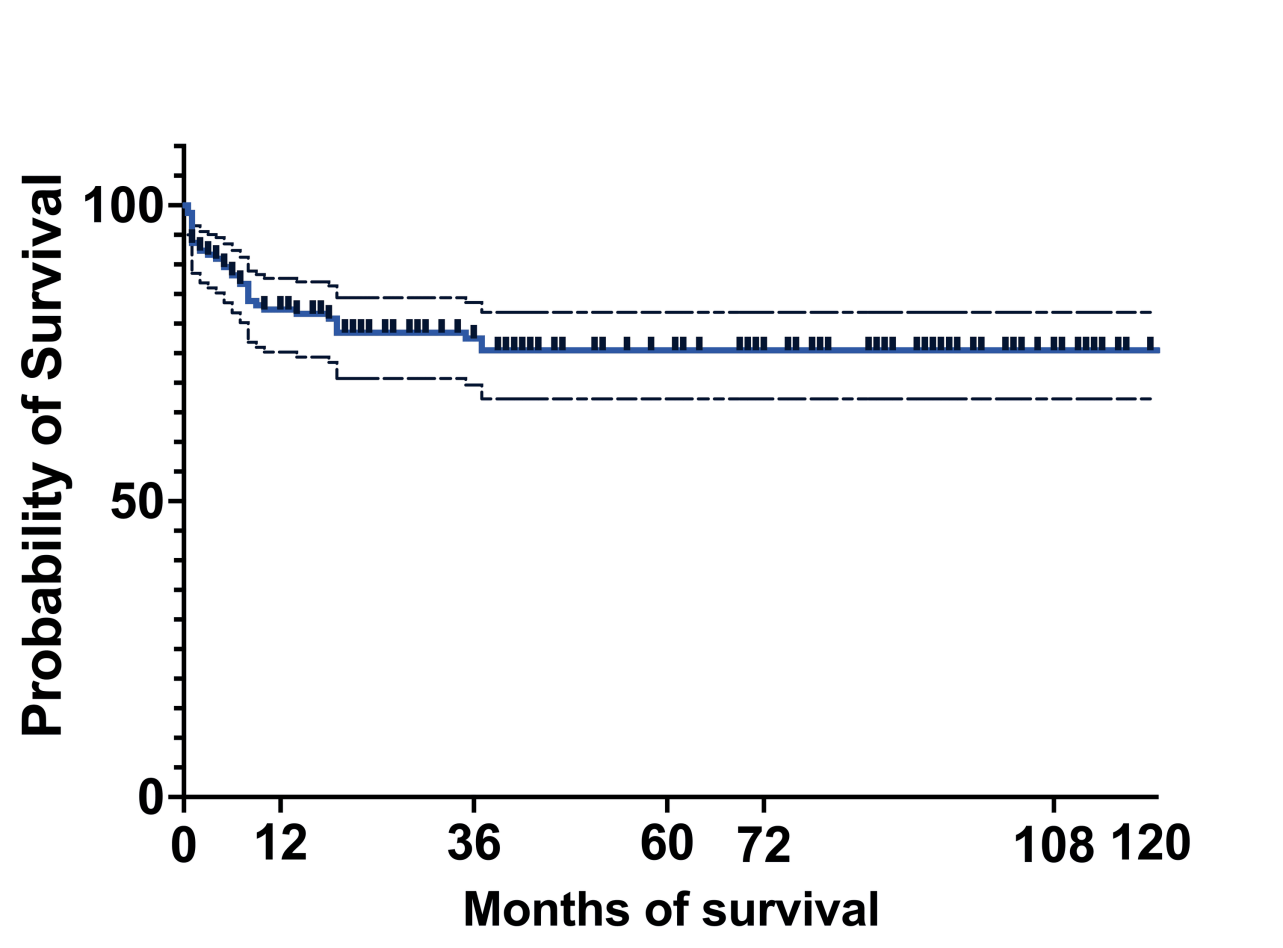
Overall survival of the entire cohort

**Figure 9:**
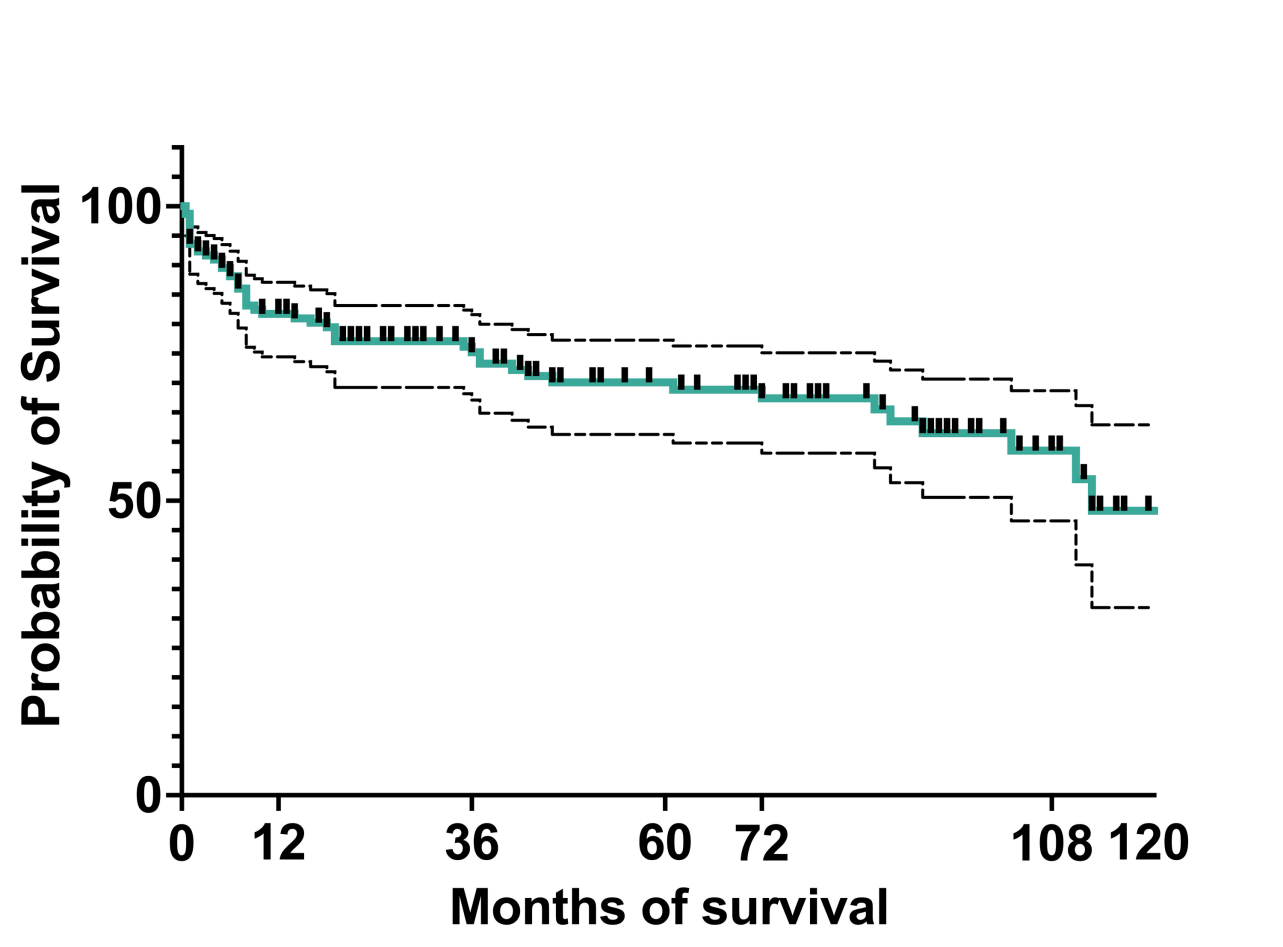
Event-free survival of the entire cohort

At 10 years, the OS of the entire cohort was 75.5% (119) (95%CI: 67.27-81.92), and EFS was 48.3% (76) (95%CI: 31.89-62.9). The median survival was 36 months, with a range of 0.5 months to 124 months (actual 95%CI: 96.36). The mean survival was 45.5 months (95%CI: 39.22-51.58) (Figures 8, 9).

**Figure 10:**
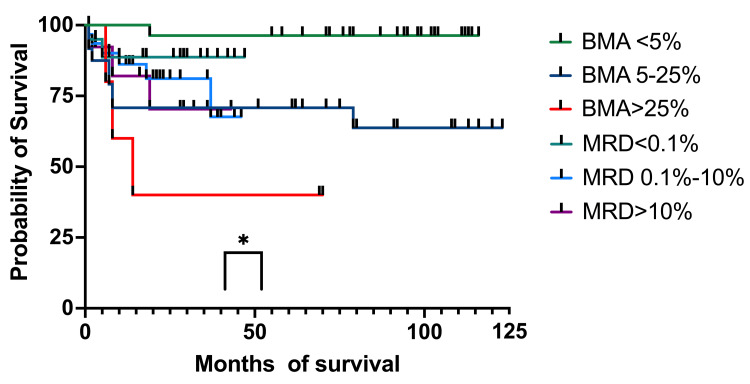
OS by BM aspirate and MRD on day 15 OS: overall survival; BM: bone marrow; MRD: minimal residual disease; BMA: bone marrow aspirate

To establish if there are any connections by survival between patients on which only BM aspiration and the percentage of blasts were determined morphologically and patients who were MRD tested on day 15, after eliminating patients who were at that moment in aplasia, we found a correlation between the two (p = 0.022 for OS and p = 0.036 for EFS) (Figure 10). We stratified MRD responses into three groups: <0.1%, 0.1%-10%, and >10%.

At one year, OS for patients with less than 5% blasts had 100% (27 patients) survival, while patients with MRD<0.1% had 88.16% (13 patients) OS survival (95%CI 79.73-100). The 5%-25% blasts group had an OS of 70.83% (19 patients) (95%CI 56.78-93.01), while the 0.1%-10% MRD group had an OS of 86.16% (22 patients) (95%CI 77.73-100). The group with more than 25% blasts had an OS of 60% (four patients) (95%CI 31.83-100), while the patients with >10% MRD had an OS of 82.05% (nine patients) (95%CI 68.81-100). At three years, OS for patients with less than 5% blasts in the BM had 96.29% (27 patients) OS (95%CI 93.12-100), while for patients with MRD below 0.1% (19 patients), the OS remained the same as at one year. For the 5%-25% blasts in the BM group, the OS was the same as the one at one year, and patients with 0.1%-10% MRD had an OS of 81.09% (17 patients) (95%CI 70.34-100). For the >25% blasts in the BM group, the OS was 40% (three patients) (95%CI 5.2-75.28), and for those with MRD of >10% (seven patients), the OS was 70.33% (95%CI 51.02-100). At four years all the percentages were the same except for the 1%-10% MRD group, which had an OS of 67.58% (two patients) (95%CI 48.28-100). At five years, the OS for the <5% blasts in the BM group and patients with MRD <0.1% the OS was the same as the ones at one, three, and four years. For the patients with 5%-25% blasts and those with MRD between 0.1%-10%, the OS was the same as one, three, and four years. For patients with >25% blasts in BM and those with MRD >10%, the OS was the same as the percentages registered at three and four years.

**Figure 11:**
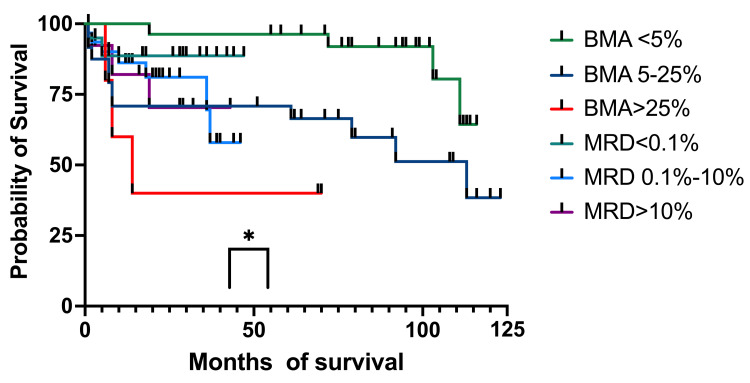
EFS by BM aspirate and MRD on day 15 EFS: event-free survival; BM: bone marrow; MRD: minimal residual disease; BMA: bone marrow aspirate

Regarding the EFS, at one year, for patients with <5% blasts in BM, it was 100% (27 patients), and for the MRD group, <0.1% was 88.66% (15 patients) (95%CI 80.25-100) (Figure 11). For patients with 5%-25% blasts in BM, the EFS was 70.83% (19 patients) (59.25-93.32), while for patients with MRD between 0.1% and 10%, the EFS was 88.16% (23 patients) (95%CI 77.73-100). The >25% blasts in the BM group had an EFS of 60% (4 patients) (95%CI 84.88-93.27), while for the MRD group with >10% abnormal immunophenotypes, the EFS was 82.05% (nine patients) (95%CI 68.81-100). At three years, patients with less than 5% blasts and those with <0.1% MRD had identical EFS as the ones at 12 months. For patients with 5%-25% blasts in BM, the EFS was identical to the one at 12 months, and the MRD 0.1%-10% group had an EFS of 69.51% (eight patients) (95% CI 51.78-100), and the group with >25% blasts in BM had the same EFS of 40% (three patients), identical to the one at 12 months. The >10% MRD patients had an EFS of 70.33% (seven patients) (95%CI 51.02-100). At four years, patients with <5% blasts in BM and the ones with MRD of <0.1% had the same EFS as the one at three years. The group with BM blasts of 5%-25% had the same EFS as the one at one and three years, while the MRD group of 0.1%-10% had an EFS of 57.92% (six patients) (95%CI 35.31-90.62). Patients with more than 25% blasts in BM and those with MRD >10% had the same EFS as the previous year. At five years, EFS was the same as at one, three, and four years for both the <5% BM blasts and MRD<0.1%. For the 5%-25% blast in the BM group and the MRD 0.1%-10% group, EFS was the same as the one at one, three, and four years. For patients who had >25% blasts in the BM, the EFS was identical to those at one, three, and four years. For patients with MRD > 10%, the EFS was identical to the ones registered at three and four years.

## Discussion

It is important to emphasize that ALL is a highly curable disease, especially in high-income countries, where CR rates as high as 80%-90% can be achieved [20]. The five-year OS for ALL is 80% in Europe and even higher in other countries. These high CR rates are attributed to the implementation of harmonized treatment protocols and better supportive therapies. However, socioeconomic status also plays an important role in long-term survival [21]. As of 2017, although Romania is classified by the International Bank for Reconstruction and Development as a high-income country [22], there are many disparities in the Romanian population, such as lack of parent education, low income, lack of employment, crowded living, and living in isolated rural areas. Our cohort was diagnosed in Cluj-Napoca at the oncology and hematology department of the Emergency Hospital for Children, which treats 7% of all cancer cases in the country. The department also treats solid tumors and non-hematological malignancies, serving the northwestern portion of the country, where the highest incidence of cancer occurs (11.25/100,000 inhabitants).

Regarding ALL, over 10 years, 15.3% of the country’s acute leukemia cases were treated in our department, with the department ranking third in Romania for treating this pathology in children [23].

The median follow-up of our patients was 44 months (3.6 years), ranging between 4.2 months and 124.3 months (10 years). This short median follow-up could be because some adolescents were redirected to adult centers at the age of 18; others may have chosen local hospitals for regular monitoring (after finishing their maintenance chemotherapy), did not show up for regular check-ups, relapsed and/or went to other centers for transplantation (local or cross-border), or died. The median OS was 45.32 months/3.8 years (standard deviation, SD=39.38 months), and the median EFS was 42.64 months/3.6 years. The survival curves of the 2011-2015 cohort revealed identical OS and EFS at five years, most likely because all patients who relapsed also died. For the 2016-2020 cohort, at four years, OS was 77% (59 patients) and EFS was 65% (50 patients), with the former being higher than that of the 2011-2015 cohort.

The mean age at diagnosis was 5.6 years, which was consistent with the literature [20,24]. The difference in numbers of male and female patients was not revelatory, as they were very close, but more girls were diagnosed with ALL. The female-to-male ratio was 1.1/1, which was lower than that reported by the National Registry of Pediatric Oncology and Hematology (RNOHP) analysis and elsewhere [23].

Within the 2011-2015 cohort, OS at five years was slightly lower for females, but EFS was higher for males. Within the 2016-2020 cohort, OS was higher for males than for females, and EFS was much lower for females than for males, implying that females suffered more events (i.e., death, relapse). Although the 95% CI was very broad for females, most likely due to the small number of cases included in the study, EFS was much higher, as most females with relapse achieved CR.

Most patients were stratified into the one- to six-year age group, as expected; 60 (40%) patients were >6 years of age, and only five (3.1%) were <1 year of age. Referring to survival by age groups, we observed that for the 2011-2015 cohort, the five-year OS was highest in the one to six-year age group, which was anticipated, followed by the >6-years and <1-year age groups. The EFS at five years was slightly lower than OS for the <1-year age group and OS was 50% for the entire study period. The 2016-2020 cohort had only one infant (<1 year of age), who died at five months. The OS at four years for the one-to-six-year age group was higher than that in the 2011-2015 cohort, whereas it was lower in the >6-year age group. The EFS at four years was lower for the one-to-six years age group but much lower for the >6-year age group, meaning the latter had the most events. For the 2016-2020 cohort, we obtained a statistical correlation for OS (p = 0.01) and EFS (p = 0.008). Therefore, the null hypothesis that the three age groups have the same survival was rejected, with the survival curves for OS and EFS depending on age.

In our study cohort, many patients had the B immunophenotype of ALL, as the T immunophenotype was found only in 23 (15%) cases. Most children, 120 (75.9%), had a normal karyotype, only 11 (7%) had the hyperdiploid or hypodiploid karyotype, and 19 (12%) had isolated other anomalies, which had no significance or were hard to interpret. The most frequently found translocation was ETV6-RUNX1, but more than half of the patients had no detectable translocations. These translocations were not analyzed in detail because they were not available since the beginning of the study, and only a few patients had a detectable fusion. The detection of more in-depth translocations such as intrachromosomal amplification of chromosome 21 (iAMP21), IGH translocations, IKZF1 gene deletions, and IKZF1plus became available in Romania in 2023 and are investigated in a single center. We found that OS at five years in the 2011-2015 subcohort was almost 80% (51 patients) for the B immunophenotype and only 44.4% (four patients) for the T immunophenotype. These results had a strong statistical correlation (p = 0.002), meaning that patients with the B immunophenotype had a higher probability of survival. Compared to OS, EFS at five years was somewhat higher for the B immunophenotype, whereas it was the same as OS for the T immunophenotype and constant across all analyzed years. Therefore, patients with the T immunophenotype did not have any events other than death, which occurred early in the first year (p = 0.04). For the 2016-2020 subcohort, OS for the B immunophenotype group was lower than that of the 2011-2015 subcohort, but it was significantly higher in the T immunophenotype group at eight (62.3%) patients. The EFS was 53% (34) for those with the B immunophenotype of ALL and 62.3% (eight) for those with the T immunophenotype, the same as OS and constant across all analyzed years. The EFS for the T immunophenotype indicated that deaths occurred early in the first year, and no other events took place after this period.

We divided the patients into three groups based on leukocyte count at diagnosis and analyzed survival for the two subcohorts. For the 2011-2015 subcohort, the five-year survival rate was 85% (39) for patients with leukocyte counts of <20,000/µL, 65% (11) for patients with leukocyte counts of 20,000-100,000/µL, and only 50% (five) for those with leukocyte counts of >100,000/µL. Patients with leukocyte counts of >100,000/µL died in the first year, and most of these patients had the T immunophenotype of ALL. The EFS was 86.4% (40) for the <20,000/µL group, 66.7% (11) for the 20,000-100,000/µL group, and 66.7% (11) for the >100,000/µL group. These results were statistically significant (p = 0.008 and p = 0.003), meaning that the null hypothesis was rejected, and the three groups had different OS and EFS curves, dependent on leukocyte count. The <20,000/µL group of the 2016-2020 cohort had a lower OS compared to that of the 2011-2015 cohort, but survival was higher for the other two groups. The EFS for the >100,000/µL group had events (i.e., death, relapse) only in the first year. The EFS was lower for patients with <20,000 leukocytes/µL and 20,000-100,000 leukocytes/µL.

In the 2011-2015 cohort, the five-year OS was 80.3% (49) for the PGR group and 50% (six) for the PPR group. We found a significant correlation between prednisone response and overall survival. The EFS at five years was the same as OS, meaning that no PGR patients suffered any other event in this time interval. At five years, only 50% of the PPR group survived. This percentage was the same as that at three years, indicating that the deaths occurred in the first three years. In the 2016-2020 cohort, the four-year OS was 78.3% (48) for the PGR group and 53% (seven) for the PPR group, the same as at three years. These percentages were somewhat higher than those of the 2011-2015 cohort, which was a statistically significant difference. The EFS was 56.4% (34) for the PGR group and 53% (seven) for the PPR group. The same interpretation is given for this cohort, where PPR patients had events only in the first three years.

Entering CR was established morphologically until 2017 and by MRD after this period. By day 15, half of the patients had entered CR morphologically, but a significant number of children (36, 23.3%) were in aplasia, making it impossible to establish CR. By day 33, 113 (78.5%) patients had entered CR, but 22 (15%) were in aplasia. Therefore, at this key moment, remission could not be verified, which was a major disadvantage, as it is of utmost importance to determine CR on day 33 before additional chemotherapy is administered. Patients diagnosed since 2017 benefitted from MRD evaluation, which is an essential tool for the evaluation of CR. By day 15, 20 (29.4%) patients had entered CR, and 17 (25%) had MRD values of 0.1%-1%. By day 33 (only available from September 2019), most patients (14, 64%), had entered CR. There is an important difference in establishing the risk groups in these two time periods: before MRD, most of the children were stratified into the MRG (31 patients, 46%) and SRG (28 patients, 39%), and only 11 (15%) patients were in the HRG. Testing for MRD changed this distribution: 40 (57%) patients were stratified into the MRG and 26 (36%) patients into the HRG. Thus, more patients received more intense chemotherapy. Before MRD testing, we observed that more patients relapsed and/or died, whereas, after the introduction of MRD testing, fewer patients relapsed and/or died, highlighting the importance of MRD. When we analyzed the survival curves of the different risk groups, for the 2011-2015 cohort, five-year OS was very high for the SRG (93%, 26 patients) and low for the HRG (27.3%, three patients). The EFS at five years was the same for the SRG (93%, 26 patients), 80.64% (25 patients) for the MRG (the same as OS and EFS at three years), and 27.3% (three patients) for the HRG (the same as the one at three years and the same as OS at three years). We found a strong statistical correlation for OS (p = 0.002) and EFS (p = 0.001). The survival curves for both OS and EFS were different, depending on the risk group stratification. Both OS and EFS did not change in the MRG and HRG from one to three years, indicating that all events took place within the first three years. For the 2016-2020 cohort, OS was constant for the SRG during the four years of follow-up (78%, 6 patients), but lower than that of the 2011-2015 cohort. At four years, in the 2016-2020 cohort, OS was 80% (32 patients) for the MRG and 66% (17 patients) for the HRG, which was higher than that of the 2011-2015 cohort, especially for the HRG. Event-free survival at four years for the SRG group was the same as OS; half of the MRG had no events (lower than the 2011-2015 cohort), and a higher percentage of the HRG group had no events.

We then analyzed the OS and EFS of patients who only had BM aspirates on day 15 and compared them to children who had MRD results on day 15. Children with aplasia were excluded from this analysis. The OS for the patients with less than 5% (96%, 27 patients) blasts and those with 5%-25% (71%, 19 patients) had a higher survival at four years compared to those with MRD <0.1% (89%, 15 patients) and MRD 0.1%-10% (68%, six patients). When we looked at those with >25% blasts and MRD >10%, we observed a higher probability of survival for the second group (40% (three patients) and 70% (seven patients), respectively). We found a statistical correlation between the OS and the percentage of blasts in BM and MRD (p = 0.033). The BM aspiration group with blasts <25% had better survival rates than those with MRD<10%, although MRD made a difference when it was >10%, with a much higher survival than of patients with >25% blasts in the BM. When looking at EFS, the results were similar to OS at four years. The only group who suffered events (relapses) was MRD (0.1%-100%) with 58% (six patients) EFS. The curve comparison is statistically significant (p = 0.036) and can be interpreted in the same way as OS.

After first-line treatment completion or during treatment, 107 (68%) patients were in CR, 21 (13%) died, and 17 (11%) relapsed. Considering the patients who relapsed, most of them were early, followed by very early relapse and late relapse. Possibly our most important result was that none of the patients with very early relapse achieved a second remission and subsequently died, whereas all patients with late relapses entered a second CR, as emphasized by the statistical correlation between the relapse groups (p = 0.0296). Of the 27 (17%) patients who eventually died, most passed after induction therapy due to toxicity or relapses, and 11 (7%) died in induction due to toxicity.

Based on the number of new cases of ALL per year, the number of new cases dropped in 2016, which was confirmed by the calculated incidence per year. Comparing these results to the RNOHP study, we observed that this decline in cases and incidence of ALL happened all over Romania in the same year. It should be noted that the constancy in the number of cases per year and the incidence rates per year are good quality indicators for data collection [23]. The exact cause of the decreased incidence of ALL cases in 2016 should be investigated further; it could be partly because many legal guardians chose to begin treatment in other countries than Romania and never went to a public onco-hematology department. The highest incidences were in 2018, 2017, 2020, and 2015. The highest crude rates were for children aged between 0 and four years (comparable with the international age group distributions), followed by patients between five and nine years of age, and finally patients between 10 and 14 years and 15 and 18 years of age. These rates were very similar, as expected.

The five-year OS was 75.3% (56 patients) for the 2011-2015 cohort, which increased by 2% for the 2016-2020 cohort. At 10 years, OS and EFS for the whole cohort were 76% (115 patients) and 48.3% (73 patients), respectively. This increase is partially due to the MRD implementation, which improved the survival in the >10% MRD group by intensification of chemotherapy. Another factor that improved survival could be better supportive care for patients with treatment-related complications. 

This study has some limitations, such as the relatively small number of patients included, which made it difficult to evaluate survival in terms of other variables. Another limitation is that there are very few studies in Romania on ALL, which prevents comparison of results with other centers. 

The strength of our study is that we evaluated in detail the cohorts by descriptive statistics, epidemiologically, and by survival rates. To our knowledge, no other center treating ALL in Romania has done such an exhaustive analysis of their patients for a long period of time.

Future studies should continue to evaluate survival for more cohorts and for longer periods of time. More variables also should be included, especially MRD testing on days 15 and 33. The socioeconomic status of patients and legal guardians should also be studied in detail to determine which factors are responsible for treatment adherence, as this is an important matter in Romania.

## Conclusions

Based on our results, the survival rates of children diagnosed with ALL in our study indicated steady improvement over time, approaching those in other developed European countries. Our two subcohorts showed an increase in survival of 2% when comparing the two time periods.

The introduction of MRD testing was a key moment in ALL diagnoses, making an important change in the stratification of risk groups. More patients were distributed in the MRG and HRG, which contributed to the use of more intense chemotherapy arms and, in some cases, hematopoietic stem cell transplantation. Patients with less than 25% blasts in BM on day 15 had a higher OS and EFS at four years than those who had <10% MRD, but the real improvement in survival rates was for the children with >10% MRD compared to patients with >25% blasts on BM aspirates (70% versus 40%).

Patients with high leukocyte counts and/or the T ALL immunophenotype suffered more events (death, relapse), but all events took place only in the first year after diagnosis.

Over the last few years, a significant increase in survival of patients with the T immunophenotype was observed. We discovered that none of the patients with very early relapse achieved CR, but all patients with late relapses were able to enter a second CR. Although female patients had more events than male patients, the former had a higher survival rate, as they mostly entered a second CR.

## Appendices

Appendix A 

**Table 8:** European standard population by age groups

Age group (years)	European standard population	Population weight
0	1 000	0,233
1-4	4000	0,256
5-9	5500	0,256
10-14	5500	0,256
15-19	5500	0,233
